# Supplementary material for: Structural basis for the oligomerization-facilitated NLRP3 activation
Source: Nat Commun. 2024 Feb 7;15:1164. doi: 10.1038/s41467-024-45396-8 (PMC10850481; doi:10.1038/s41467-024-45396-8)
Supplement: Supplementary file 1 — Supplementary Information [file 41467_2024_45396_MOESM1_ESM.pdf]

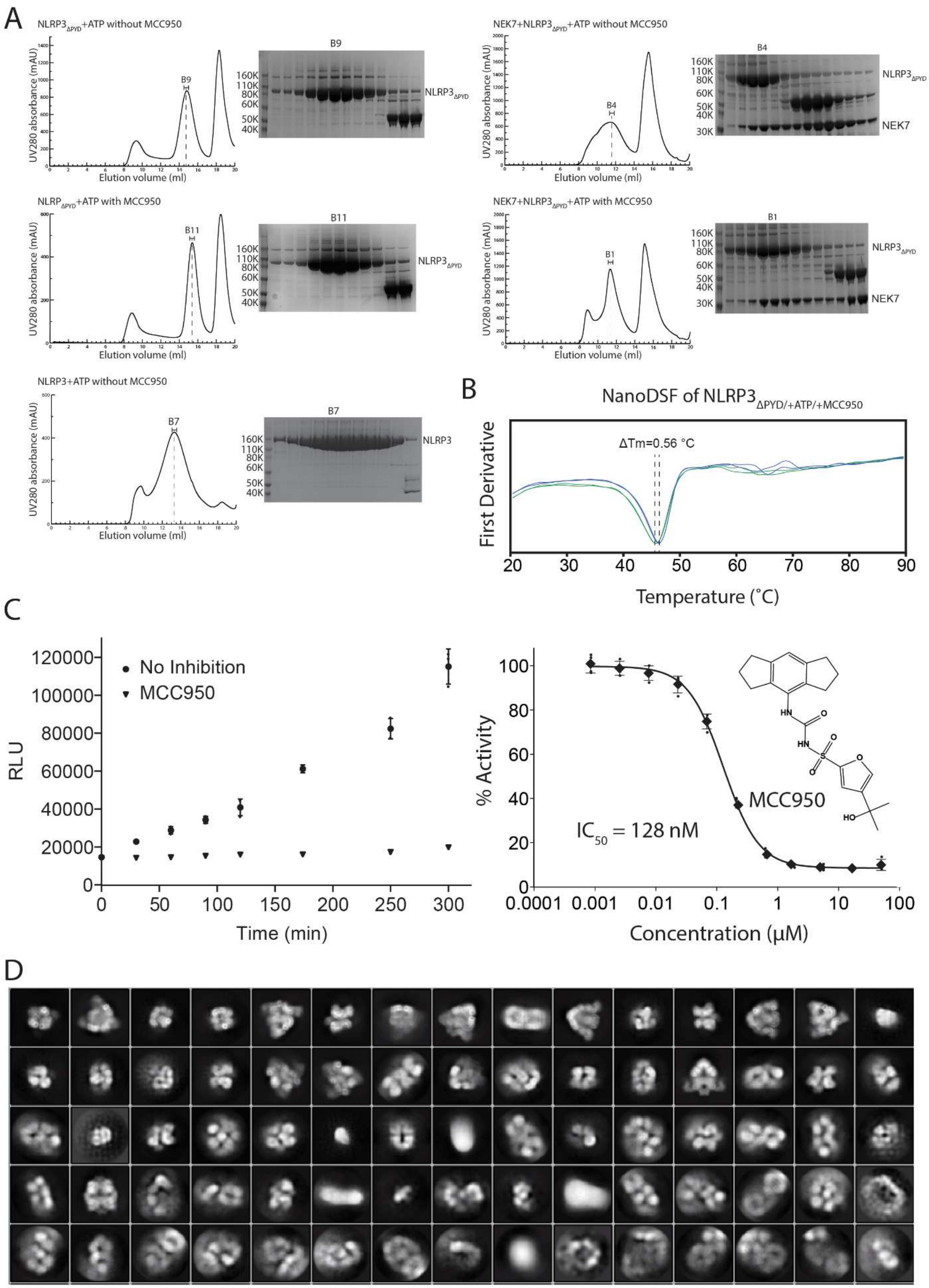
A
NLRP3ΔPYD+ATP without MCC950
B9
NLRP3ΔPYD
UV280 absorbance (mAU)
Elution volume (ml)
160K
110K
80K
60K
50K
40K
NEK7+NLRP3ΔPYD+ATP without MCC950
B4
NEK7
30K
NLRPΔPYD+ATP with MCC950
B11
NEK7+NLRP3ΔPYD+ATP with MCC950
B1
NLRP3+ATP without MCC950
B7
NLRP3
B
NanoDSF of NLRP3ΔPYD/+ATP/+MCC950
ΔTm=0.56 °C
First Derivative
Temperature (˚C)
C
No Inhibition
MCC950
RLU
Time (min)
% Activity
Concentration (μM)
IC50 = 128 nM
HN
HO
D

Supplementary Figure 1. Protein purification, thermo shift, ATP hydrolysis assay. A. Size-exclusion chromatography of NLRP3$_{\Delta PYD}$ and its complex with/without MCC950 or NEK7, and FL NLRP3 without MCC950. The fractions were examined by SDS-polyacrylamide gel electrophoresis (SDS-PAGE). Source data are provided as a Source Data file. **B.** Representative melting curves of purified NLRP3$_{\Delta PYD}$ complex in the presence of DMSO or ATP and MCC950. The melting temperatures (Tm) were indicated in the figure. **C.** Residual ATP hydrolysis activity of NLRP3$_{\Delta PYD}$ without (Left) and with (Right) MCC950. These experiments were repeated more than three times. Source data are provided as a Source Data file. **D.** Initial 2D classification results for the NLRP3$_{\Delta PYD/+ATP/-MCC950}$ dataset.

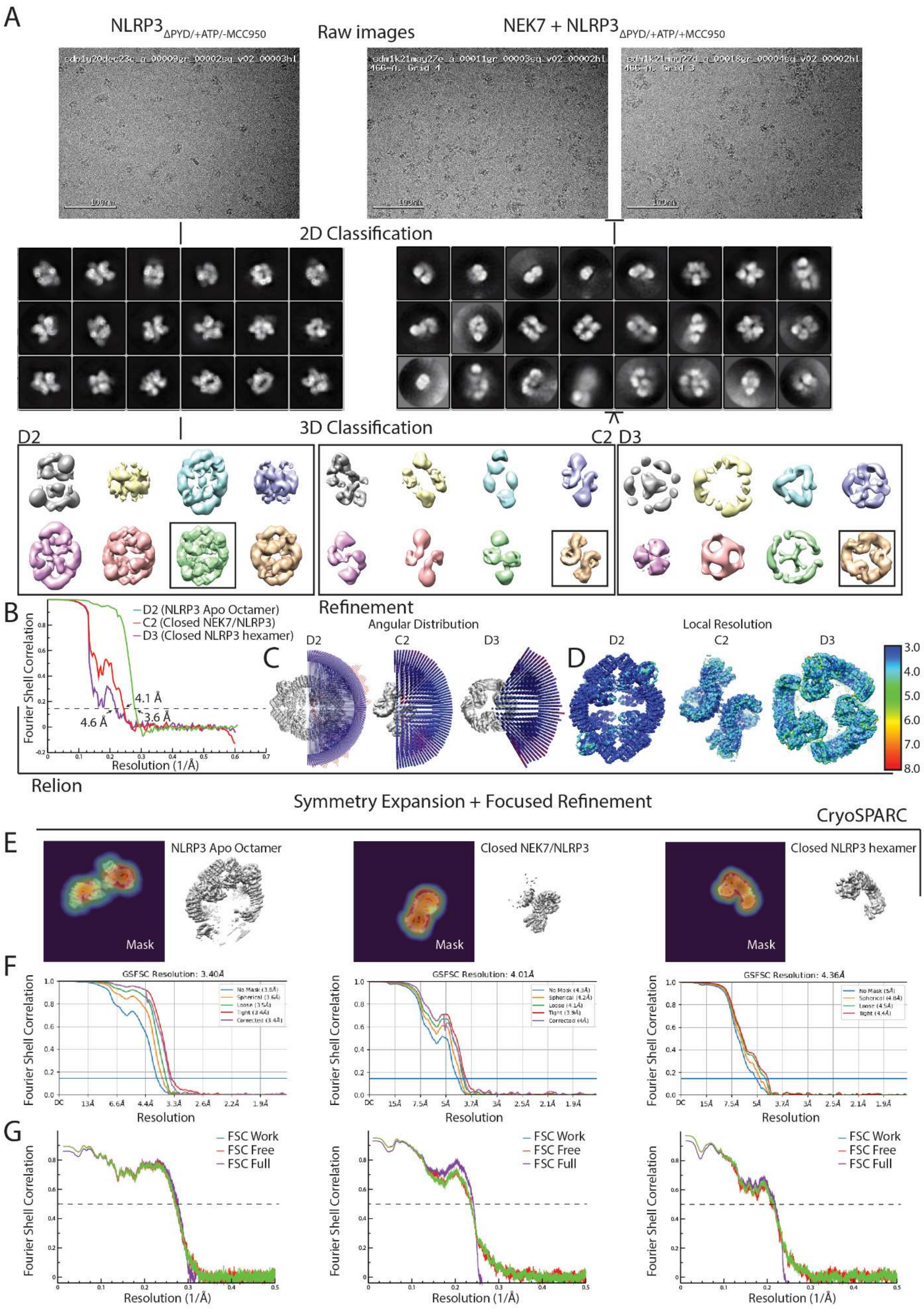

A
NLRP3 ΔPYD/+ATP/-MCC950
Raw images
NEK7 + NLRP3 ΔPYD/+ATP/+MCC950
2D Classification
3D Classification
D2
C2 D3
B
D2 (NLRP3 Apo Octamer)
C2 (Closed NEK7/NLRP3)
D3 (Closed NLRP3 hexamer)
Fourier Shell Correlation
4.1 Å
4.6 Å
3.6 Å
Resolution (1/Å)
Relion
Refinement
Angular Distribution
C
D
Local Resolution
Symmetry Expansion + Focused Refinement
CryoSPARC
E
NLRP3 Apo Octamer
Closed NEK7/NLRP3
Closed NLRP3 hexamer
Mask
F
GSFSC Resolution: 3.40Å
GSFSC Resolution: 4.01Å
GSFSC Resolution: 4.36Å
Resolution
G
FSC Work
FSC Free
FSC Full

Supplementary Figure 2. Cryo-EM analysis of NLRP3$_{\Delta PYD/+ATP/-MCC950}$, and NLRP3$_{\Delta PYD/+ATP/+MCC950}$ with/without NEK7. A. Flow chart of the cryo-EM data processing procedure. Details can be found in the Materials and methods. Representative cryo-EM micrographs were shown. **B.** Fourier shell correlation (FSC) curves of the structures with FSC as a function of resolution using Relion outputs. **C.** Angular orientation distribution of the particles used in the final reconstruction. The particle distribution is indicated by different color shades. **D.** Local resolution of the map estimated using the ResMap program and colored as indicated. **E.** Symmetry Expansion and Focused Refinement within CryoSPARC. **F.** FSC curves of the structures reported from CryoSPARC. **G.** Model validation. Comparison of the FSC curves between model and half map 1 (work), model and half map 2 (free), and model and full map are plotted in green, red, and magenta, respectively.

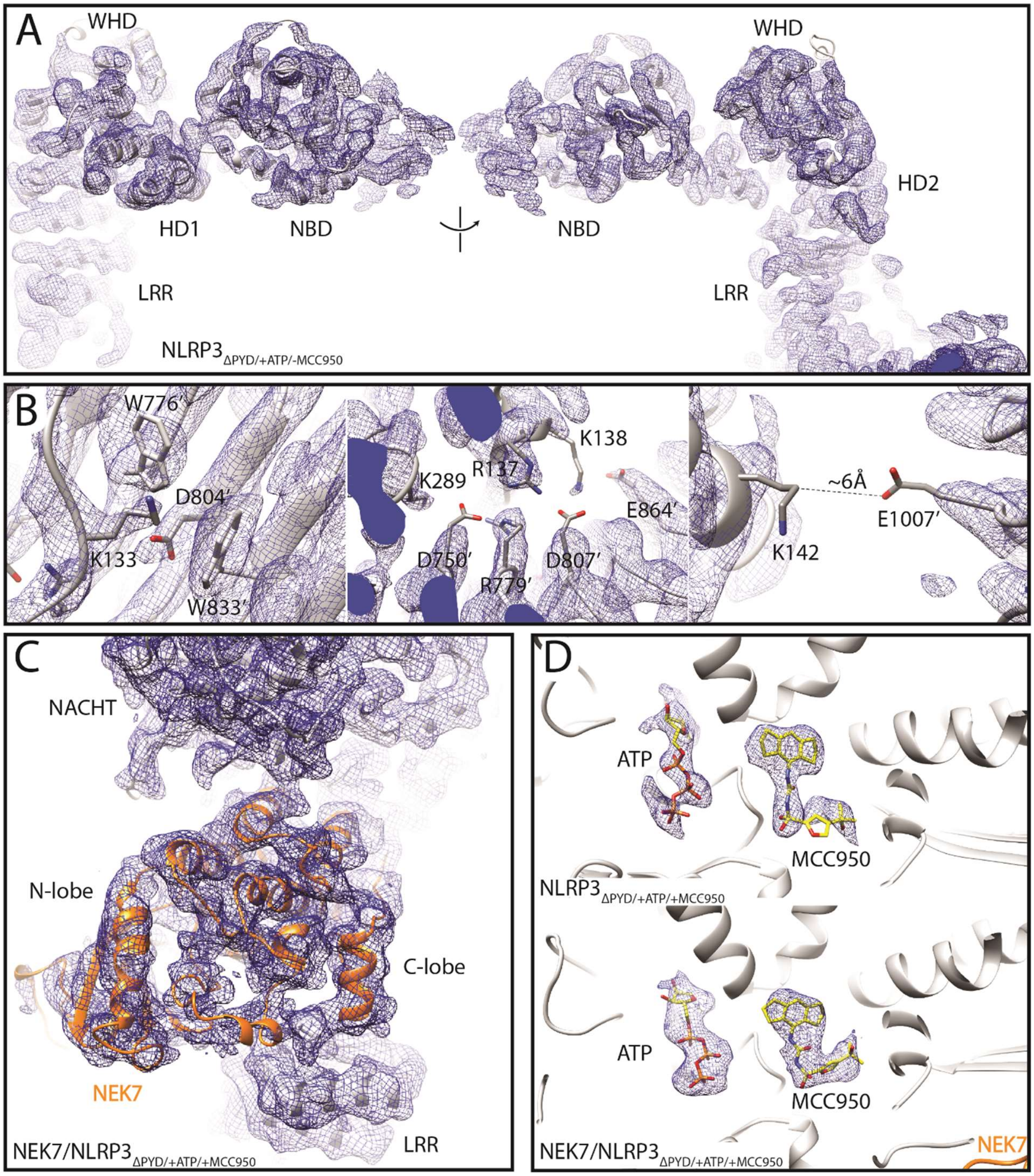

Supplementary Figure 3. Cryo-EM densities of NLRP3$_{\Delta PYD/+ATP/-MCC950}$ around NACHT (A), Head-Face interface (B), NLRP3$_{\Delta PYD/+ATP/+MCC950}$ with/without NEK7 around NEK7 (C), and ATP, MCC950 (D). Cryo-EM density is displayed as mesh at the contour level 4σ for the highlighted regions. The protein structure models, and small molecules (ATP and MCC950) were shown as cartoon and yellow sticks, respectively. NEK7 was in orange.

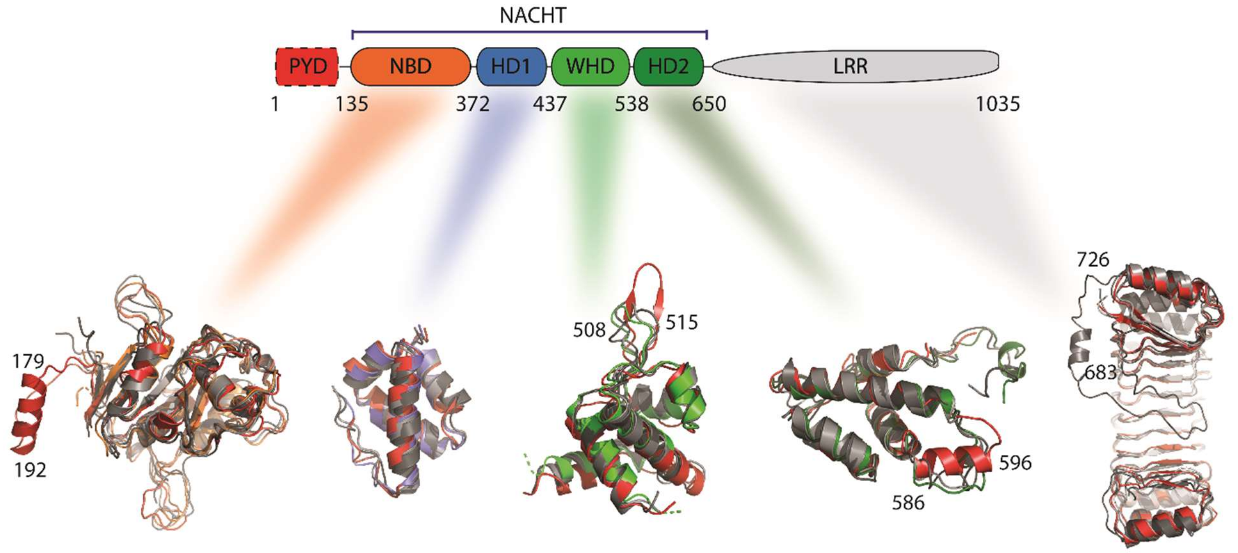

Supplementary Figure 4. Subdomain structural comparisons. NLRP3 subdomains (NBD, HD1, WHD, HD2, and LRR) from the closed cage (in gray, PDB ID: 7PZC), open cage (color-coded as diagram above, this study), and activated NLRP3 inflammasome (in red, PDB ID: 8EJ4) were superimposed. Highlighted regions (α-helix$_{179\text{-}192}$, α-helix$_{586\text{-}596}$, and hairpin loop$_{508\text{-}515}$) from the activated NLRP3 are newly formed during inflammasome assembly, while the acidic loop$_{683\text{-}726}$ from the closed cage is also highlighted.

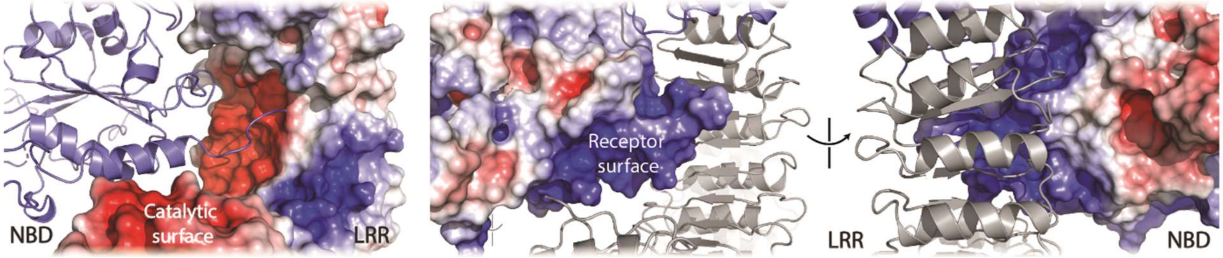

Supplementary Figure 5. Surface charge distribution at the Head-Face interfaces in NLRP3$_{\Delta PYD/+ATP/-MCC950}$ octamer. Surface charge distribution pattern of catalytic or receptor surfaces with the counterparts shown in cartoon, from the open NLRP3$_{\Delta PYD/+ATP/-MCC950}$ octamer structure. The NACHT, and LRR are colored slate, and gray, respectively.

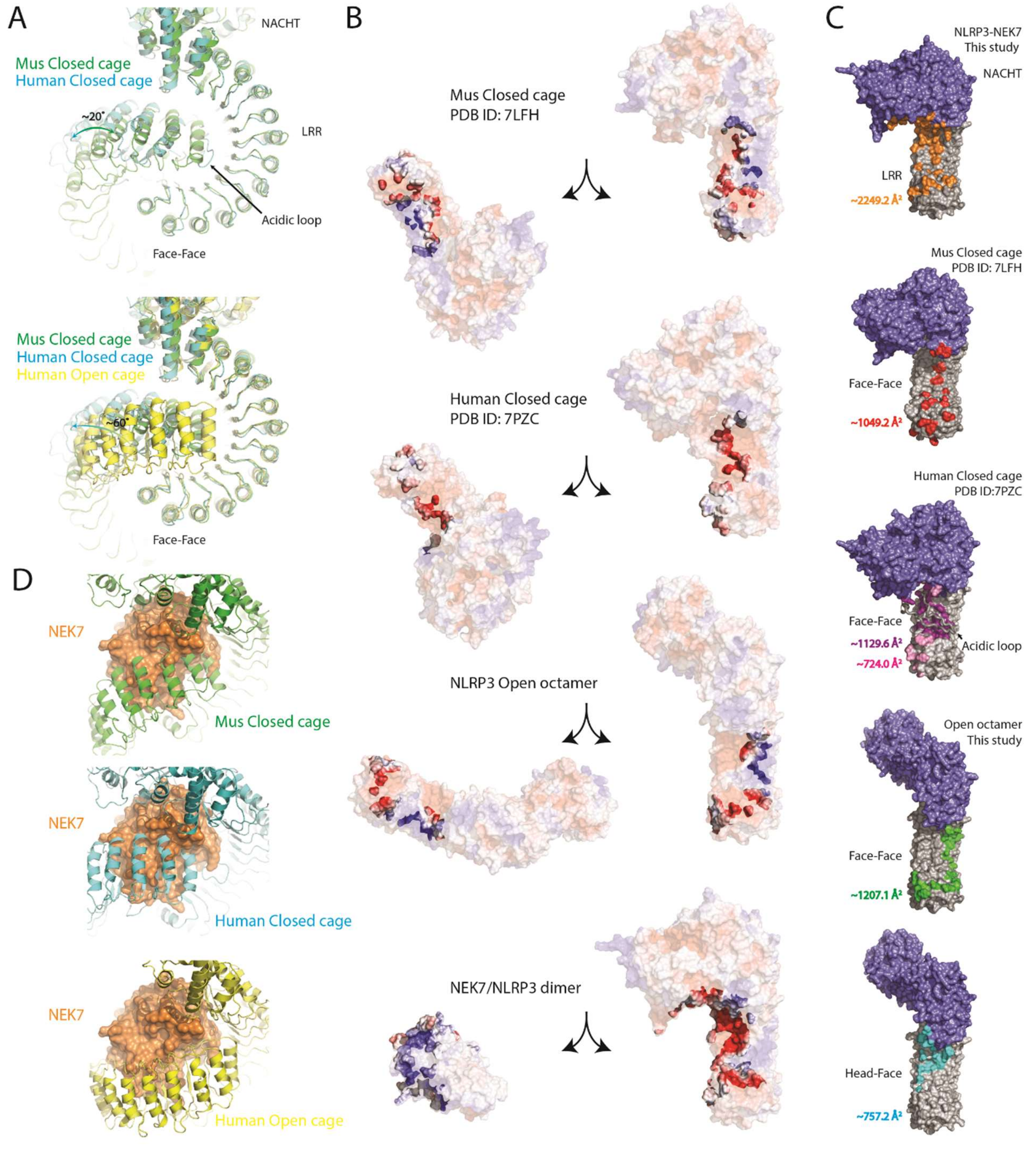

Supplementary Figure 6. Structural analysis of the LRR concave side in closed and open NLRP3 oligomers. A. Structural overlays of LRRs from FL mouse (green, PDB ID: 7LFH) and human (cyan, PDB ID: 7PZC) closed cages (top) and human NLRP3$_{\Delta PYD/+ATP/-MCC950}$ open octamer (yellow, this study, bottom). Angular deviations between human and mouse closed cages and between human closed and open cages were illustrated with curved arrows. **B.** Surface charge distribution patterns of LRR concave side surfaces from the mouse, human FL NLRP3 closed cages, open NLRP3$_{\Delta PYD/+ATP/-MCC950}$ octamer, and NEK7/NLRP3$_{\Delta PYD/+ATP/-MCC950}$ dimer structures. **C.** Top to bottom surface representations of NLRP3 in the NEK7 binding, mouse and

human closed cages, and open octamer states with concave interaction residues highlighted in orange, red, magenta, green, and cyan. NACHT and LRR were in slate and gray, respectively. The interface areas ($Å^2$) were highlighted and colored, respectively. Note: Magenta for acidic loop (cartoon) and pink for Face-Face interaction in human closed cage. **D.** From top to bottom, docking NEK7 onto the LRR concave side in mouse, human closed cages, and open octamer states.

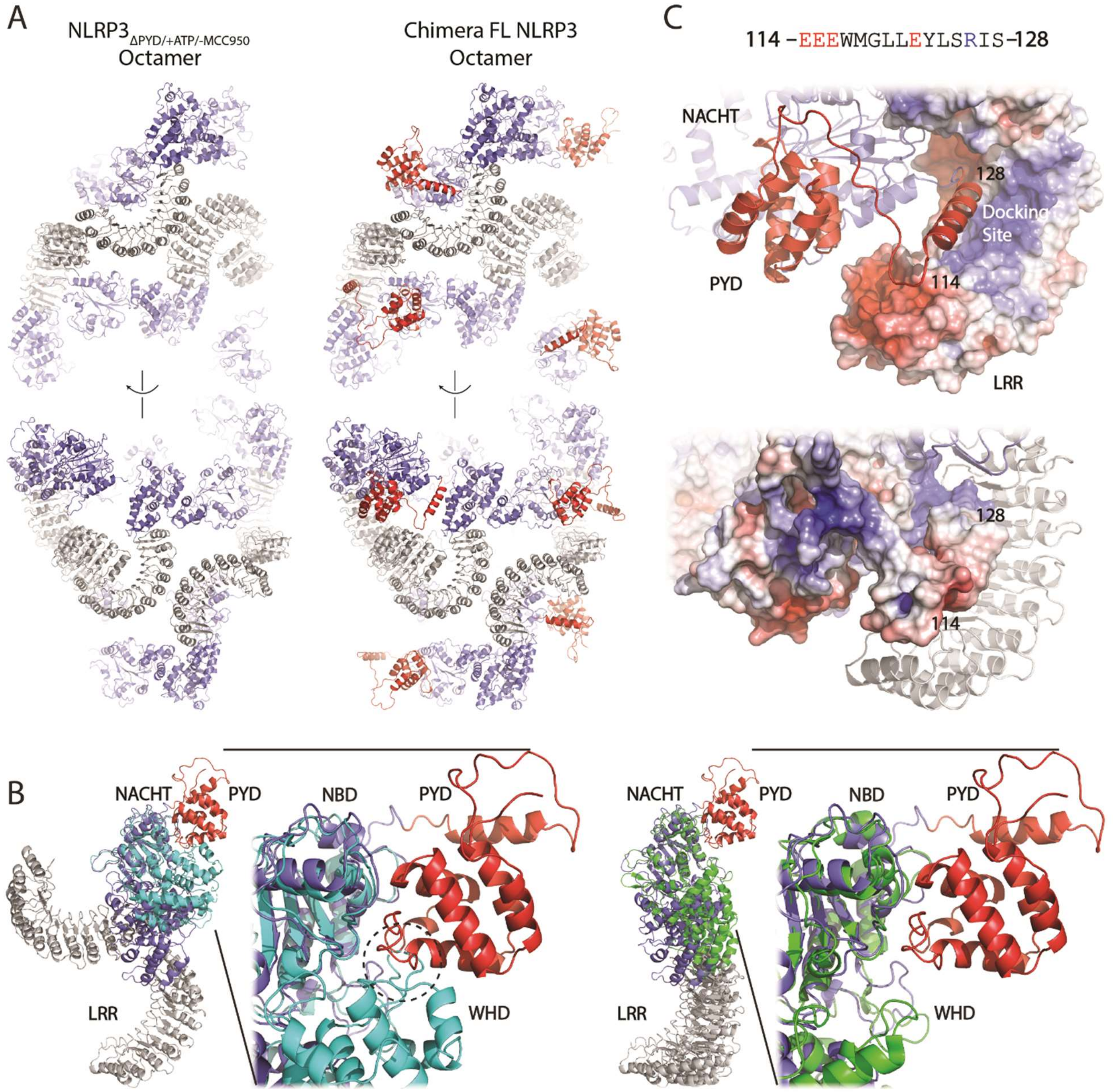

Supplementary Figure 7. Proposed PYD position in NLRP3$_{\Delta PYD/+ATP/-MCC950}$ octamer. A. NLRP3$_{\Delta PYD/+ATP/-MCC950}$ structure and FL AF2 NLRP3 chimera model in the octamer states were shown as cartoon with the PYD, NACHT, and LRR in red, slate, and gray, respectively. **B.** Putative PYD binding site on the open/activated NLRP3. Left, structural overlay of closed NLRP3$_{\Delta PYD/+ATP/+MCC950}$ and open PYD-NLRP3$_{\Delta PYD/+ATP/-MCC950}$ chimera model after MD simulation, using NBD domain as a reference. The dashed circle indicated the clashes between the PYD model and closed NACHT (in cyan). Right, structural overlay of activated FL NLRP3 (PDB ID: 8EJ4) and open PYD-NLRP3$_{\Delta PYD/+ATP/-MCC950}$ chimera model after MD simulation, using NBD domain as a reference. The NACHT in closed, open, and activated NLRP3 is colored cyan, slate, and green, respectively, while the PYD and LRR are red and gray, respectively. **C.** Proposed

docking site for the α-helix$_{114-128}$ on the catalytic LRR in the open octamer state. Surface charge distribution pattern of the catalytic protomer (top panel), and α-helix$_{114-128}$ (lower panel) were shown with the counterparts shown in cartoon. The sequence of α-helix$_{114-128}$ was shown above**.**

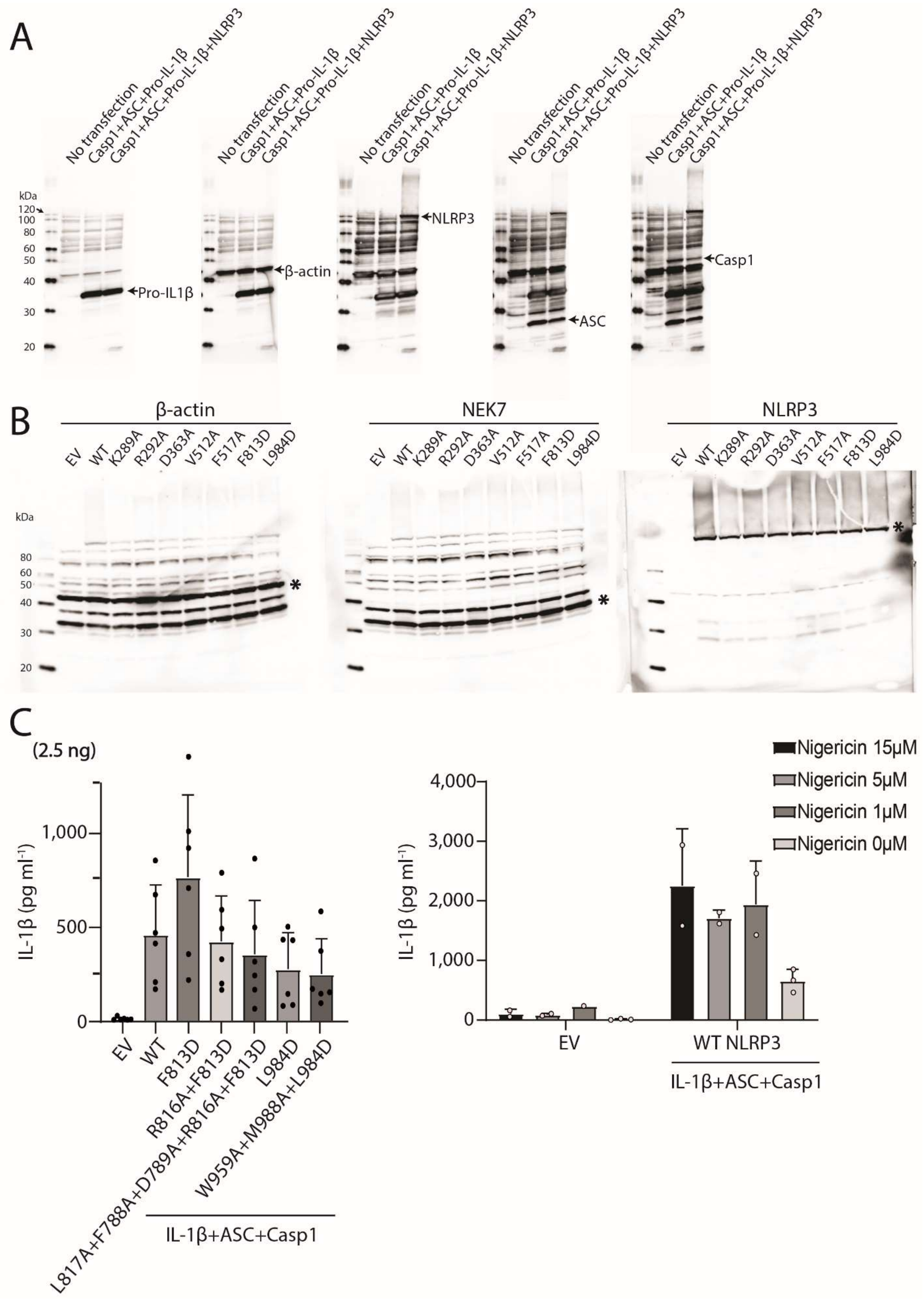
A
No transfection
Casp1+ASC+Pro-IL-1β
Casp1+ASC+Pro-IL-1β+NLRP3
kDa
120
100
80
60
50
40
30
20
Pro-IL1β
β-actin
NLRP3
ASC
Casp1
B
β-actin
NEK7
NLRP3
EV
WT
K289A
R292A
D363A
V512A
F517A
F813D
L984D
*
C
(2.5 ng)
IL-1β (pg ml-1)
1,000
500
0
EV
WT
F813D
R816A+F813D
L817A+F788A+D789A+R816A+F813D
L984D
W959A+M988A+L984D
IL-1β+ASC+Casp1
Nigericin 15μM
Nigericin 5μM
Nigericin 1μM
Nigericin 0μM
4,000
3,000
2,000
1,000
WT NLRP3

Supplementary Figure 8. Cellular assay with mutations at the human NLRP3 open octamer interfaces. A. The Western blot analysis indicated the expression levels of target proteins upon transfection of the plasmids (1 ng Casp1, 0.3 ng ASC, and 50 ng pro-IL-1β, with/without 10 ng of NLRP3), comparing to the endogenous protein β-actin. **B.** The Western blot analysis showed that transfecting NLRP3 (wild-type or carrying a point mutation) had no effect on the expression levels of β-actin, NEK7, or NLRP3. Positions of β-actin, NEK7, and NLRP3 were highlighted using stars. **C.** Left, cellular assay showing induction of IL-1β signaling upon transfection with 2.5 ng of WT or mutant FL NLRP3 vectors. Right, cellular assay showing induction of IL-1β signaling upon transfection with 2.5 ng of WT FL NLRP3 vector with various concentrations of Nigericin. These experiments were repeated more than twice. EV, empty vector control. Source data are provided as a Source Data file.

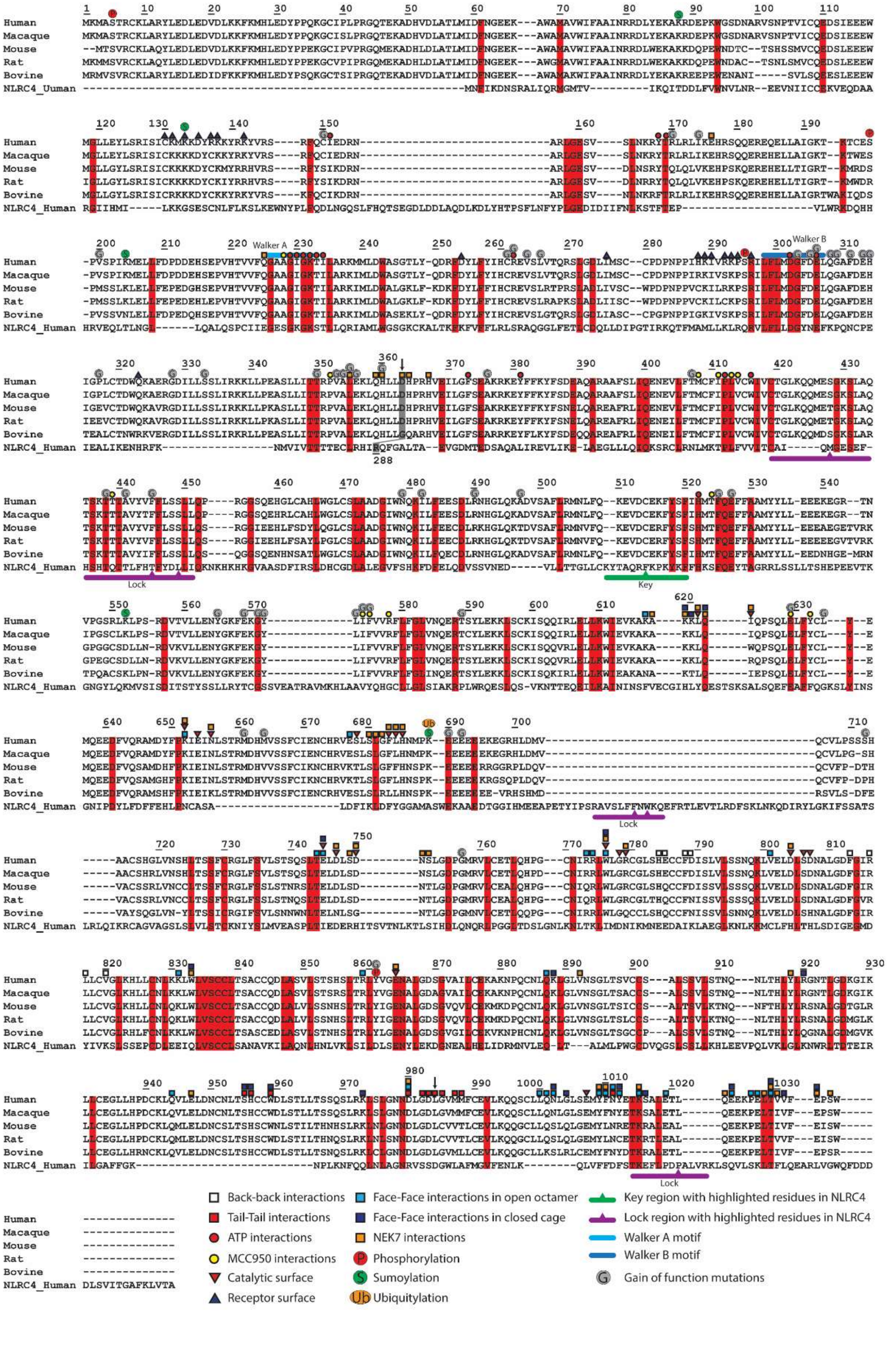
Human
Macaque
Mouse
Rat
Bovine
NLRC4_Human
Walker A
Walker B
Lock
Key
Back-back interactions
Tail-Tail interactions
ATP interactions
MCC950 interactions
Catalytic surface
Receptor surface
Face-Face interactions in open octamer
Face-Face interactions in closed cage
NEK7 interactions
Phosphorylation
Sumoylation
Ubiquitylation
Key region with highlighted residues in NLRC4
Lock region with highlighted residues in NLRC4
Walker A motif
Walker B motif
Gain of function mutations

Supplementary Figure 9. NLRP3 whole sequence alignment. The sequences have been aligned using ClustalW. Residues involved in the substrate bindings, oligomerization, NEK7 binding, and activation are highlighted. Conserved residues are highlighted and filled in red.

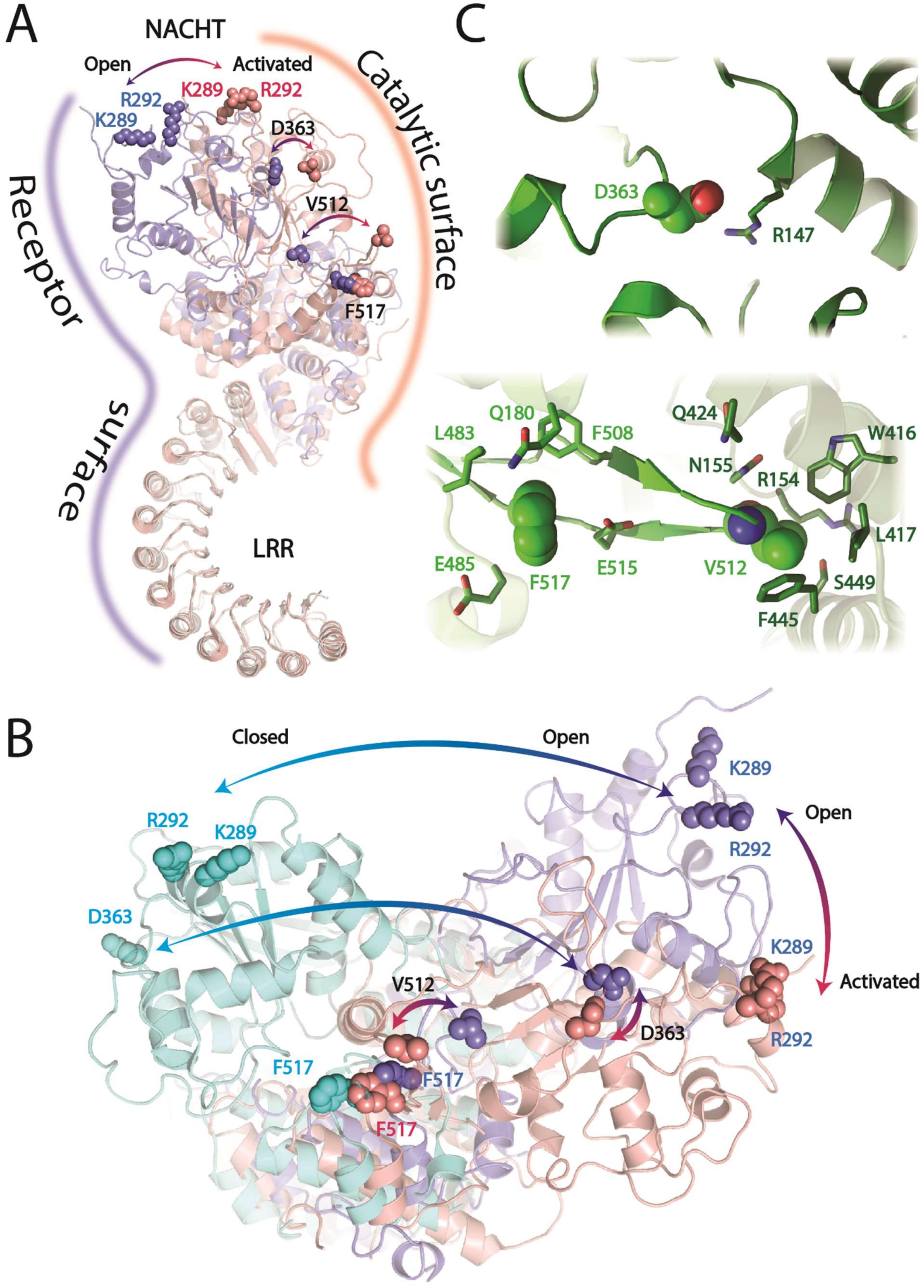
A
NACHT
Open
Activated
R292
K289
K289
R292
D363
V512
F517
Catalytic surface
Receptor
surface
LRR
C
D363
R147
Q180
Q424
L483
F508
W416
N155
R154
L417
E485
F517
E515
V512
S449
F445
B
Closed
Open
K289
R292
K289
Open
R292
D363
K289
V512
Activated
D363
R292
F517
F517
F517

Supplementary Figure 10. NLRP3 interface rearrangement upon activation. A. Structural overlay of open NLRP3$_{\Delta PYD/+ATP/-MCC950}$ to activated FL NLRP3 (PDB ID: 8EJ4) using LRR as the reference. The NLRP3$_{\Delta PYD/+ATP/-MCC950}$ and FL NLRP3 were shown as cartoon and colored in slate and gray for NLRP3$_{\Delta PYD/+ATP/-MCC950}$, and pink for FL NLRP3, respectively. The catalytic, and receptor surfaces during inflammasome assembly were highlighted. **B.** Structural overlay of activated FL NLRP3 (PDB ID: 8EJ4), open NLRP3$_{\Delta PYD/+ATP/-MCC950}$ to the closed NLRP3$_{\Delta PYD/+ATP/+MCC950}$ using LRR as the reference. The NACHT domains were in pink, cyan, or slate for the activated, closed, or open states, respectively. **C.** Zoom-in views at Asp$_{363}$, and Val$_{512}$ from the activated NLRP3 inflammasome structure (PDB ID: 8EJ4). The activator and the receptor protomers were in light, and dark green, respectively. The selected mutational residues (Fig. 4) and surround key residues were shown as spheres, and sticks, respectively. The protein structures were shown as cartoon. Subdomain reorganizations upon activation were highlighted by arrows.

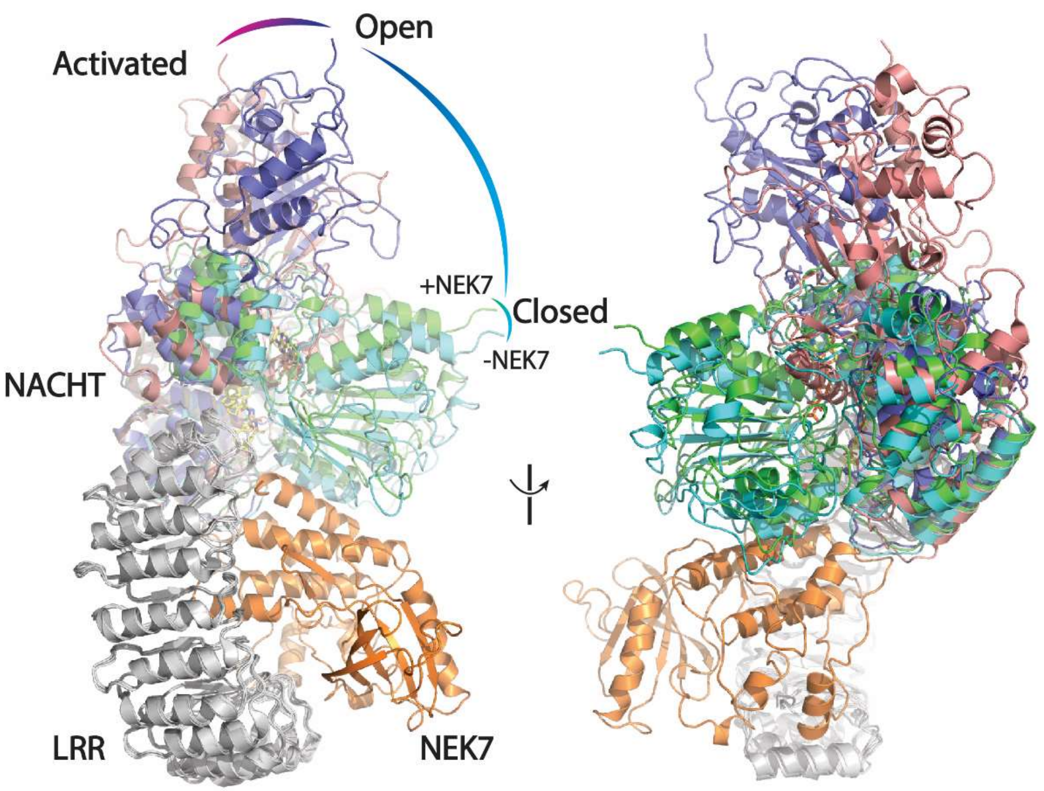

Supplementary Figure 11. NEK7 engagement induces NLRP3 conformational changes. Structural overlay of activated FL NLRP3 (PDB ID: 8EJ4) open NLRP3$_{\Delta PYD/+ATP/-MCC950}$, closed NLRP3$_{\Delta PYD/+ATP/+MCC950}$, and NEK7/NLRP3$_{\Delta PYD/+ATP/+MCC950}$ using LRR as the reference. Two different views were shown. Structural changes were highlighted by arrows. LRR, and NEK7 were in gray, and orange, respectively. The NACHT from the activated FL NLRP3, open NLRP3$_{\Delta PYD/+ATP/-MCC950}$, closed NLRP3$_{\Delta PYD/+ATP/+MCC950}$, and NEK7/NLRP3$_{\Delta PYD/+ATP/+MCC950}$ were in pink, slate, cyan, and green, respectively. The protein structures were shown as cartoon. Subdomain reorganizations upon activation were highlighted by curves.

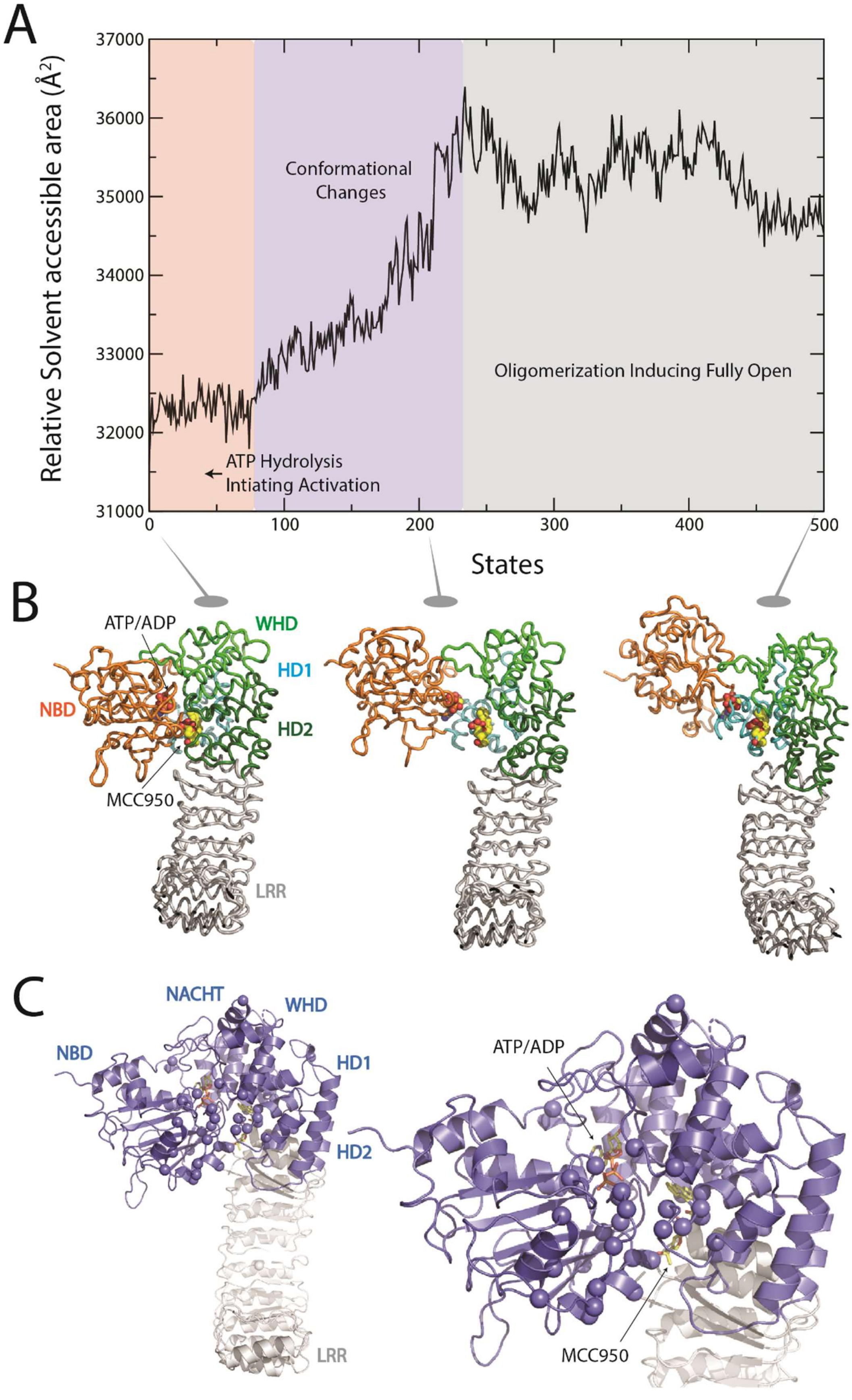
A
Relative Solvent accessible area (Å²)
37000
36000
35000
34000
33000
32000
31000
0
100
200
300
400
500
States
Conformational Changes
Oligomerization Inducing Fully Open
ATP Hydrolysis Intiating Activation
B
ATP/ADP
WHD
HD1
NBD
HD2
MCC950
LRR
C
NACHT
WHD
NBD
HD1
HD2
LRR
ATP/ADP
MCC950

Supplementary Figure 12. Target Molecular Dynamics (MD) simulations. A. Relative solvent accessible area calculated from the simulation ensembles. The relative solvent-accessible area remains stable for the first ~80 states, then increases from states 80-230, possibly due to ATP hydrolysis, solvent penetration, and catalytic scaffolding from the neighboring LRR concave side. However, it decreased thereafter, possibly due to oligomerization-induced full opening (Movie 4). **B.** Snapshots of ensembles at states 0, 200, and 487, respectively. NLRP3 was shown in cartoon and followed the color code in Fig. S4. ATP/ADP, and MCC950 were shown in ball-and-sticks serving as reference points to determine their respective positions. **C.** Global (left) and Zoomed-in (right) views of GOF mutations in spheres mapped to the closed NLRP3 structure with ATP and MCC950 shown as sticks. This suggests that GOF mutations accumulates at the NBD-HD1-WHD-HD2 interfaces near the nucleotide/MCC950 binding sites.

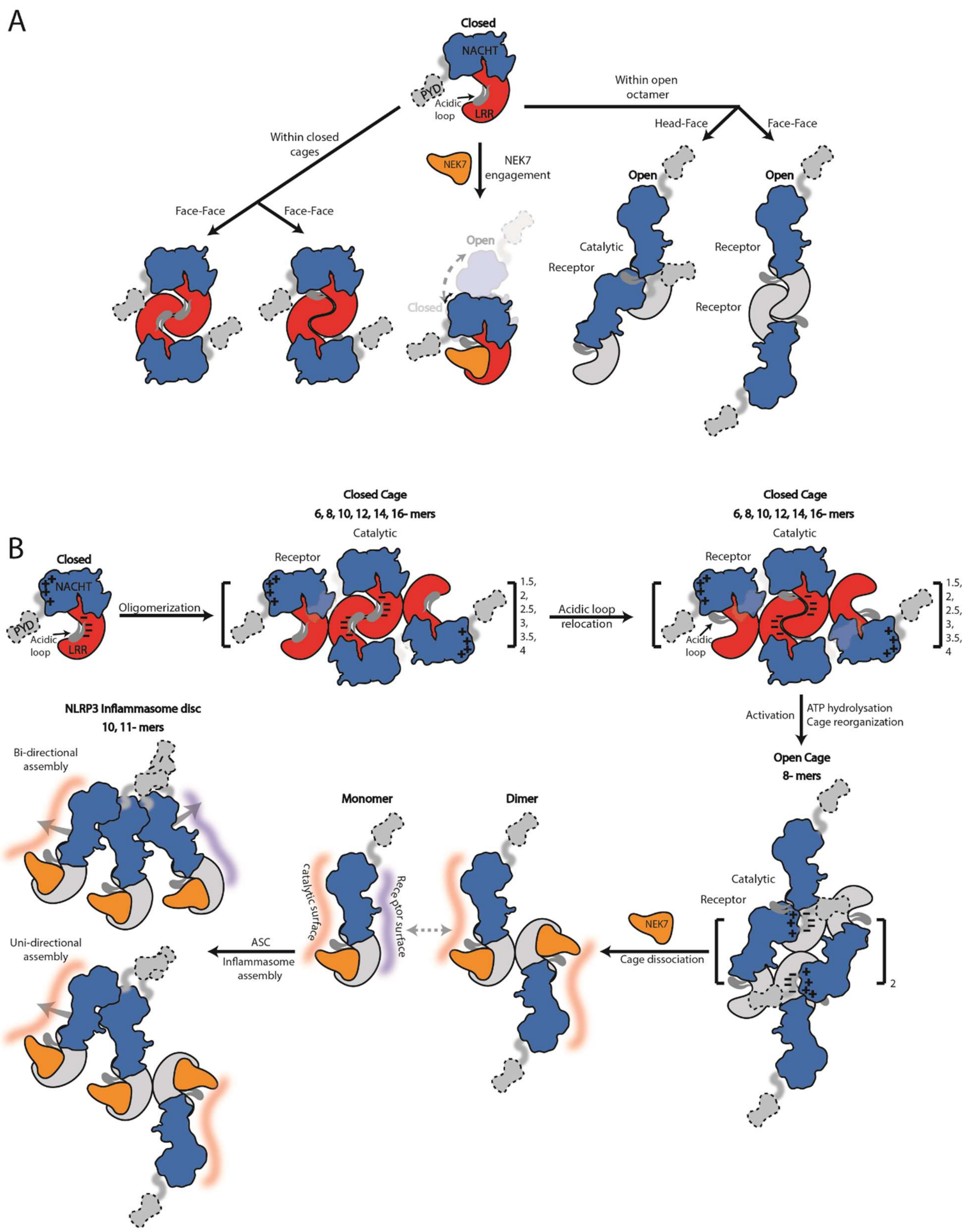
A
Closed
NACHT
PYD
Acidic loop
LRR
Within closed cages
Face-Face
Face-Face
NEK7
NEK7 engagement
Open
Closed
Within open octamer
Head-Face
Face-Face
Open
Catalytic
Receptor
Open
Receptor
Receptor
B
Closed
NACHT
PYD
Acidic loop
LRR
Oligomerization
Closed Cage
6, 8, 10, 12, 14, 16- mers
Catalytic
Receptor
1.5, 2, 2.5, 3, 3.5, 4
Acidic loop relocation
Closed Cage
6, 8, 10, 12, 14, 16- mers
Catalytic
Receptor
Acidic loop
1.5, 2, 2.5, 3, 3.5, 4
Activation
ATP hydrolysation
Cage reorganization
Open Cage
8- mers
Catalytic
Receptor
2
NEK7
Cage dissociation
Dimer
Monomer
catalytic surface
Receptor surface
ASC
Inflammasome assembly
NLRP3 Inflammasome disc
10, 11- mers
Bi-directional assembly
Uni-directional assembly

Supplementary Figure 13. Schematic representation of the proposed acidic loop’s effect on the NACHT activation. A. Acidic loop displacement in closed and open/activated NLRP3 oligomers. In closed cages (Left), the acidic loop can be at the Face-Face interface (PDB ID: 7PZC) or displaced by neighboring LRR from its concave side location (PDB ID: 7LFH, 7VTQ). In the open octamer (Right), the acidic loop can be relocated by neighboring NACHT (Head-Face interaction) or LRR (Face-Face interaction) (this study). NEK7 binding (Middle) on the concave side of LRR relocates the acid loop (this study, and PDB ID: 8EJ4). NEK7 binding subtly opens closed NLRP3 (Fig. S11). **B.** Two types of closed cages can exist: one with the acidic loop inside the Face-Face interface and the other with the loop pushed out from the concave side of LRR. We hypothesize that closed cages with the acidic loop in the Face-Face interface may form first, as the acidic loop's position is closer to the initial NLRP3 monomer configuration. Replacing the acidic loop may aid in cage reorganization and NACHT opening. NEK7 presence can dissociate the open cage into NEK7/NLRP3 monomers or dimers, allowing inflammasome formation with an odd number (11- mers) of protomers derived from even-numbered protomer closed/open cages. The catalytic, and receptor surfaces during inflammasome assembly were highlighted.

Supplementary Table 1. Cryo-EM data collection, refinement and validation statistics

<table>
<tr><th></th><th>NLRP3$_{\Delta PYD/+ATP/-MCC950}$
(EMDB-40811)
(PDB 8SWF)</th><th>NEK7/NLRP3$_{\Delta PYD/+ATP/+MCC950}$
(EMD- 40855)
(PDB 8SXN)</th><th>NLRP3$_{\Delta PYD/+ATP/+MCC950}$
(EMD-40820)
(PDB 8SWK)</th></tr>
<tr><td>Data collection and processing</td><td></td><td></td><td></td></tr>
<tr><td>Magnification</td><td>105,000 x</td><td colspan="2">105,000 x</td></tr>
<tr><td>Voltage (kV)</td><td>300</td><td colspan="2">300</td></tr>
<tr><td>Electron exposure (e–/Å^2)</td><td>53.3</td><td colspan="2">51.3</td></tr>
<tr><td>Defocus range (μm)</td><td>-1.4 to -1.9</td><td colspan="2">-1.2 to -1.8</td></tr>
<tr><td>Pixel size (Å)</td><td>0.82</td><td colspan="2">0.832</td></tr>
<tr><td>Symmetry imposed</td><td>D2</td><td>C2</td><td>D3</td></tr>
<tr><td>Initial particle images (no.)</td><td>3,758,000</td><td colspan="2">1,243,000</td></tr>
<tr><td>Final particle images (no.)</td><td>466,664</td><td>199,924</td><td>72,861</td></tr>
<tr><td>Map resolution (Å)</td><td>3.39</td><td>4.04</td><td>4.32</td></tr>
<tr><td>FSC threshold</td><td></td><td></td><td></td></tr>
<tr><td>Map resolution range (Å)</td><td>3.3-8.0</td><td>4.0-8.0</td><td>4.3-8.0</td></tr>
<tr><td>Refinement</td><td></td><td></td><td></td></tr>
<tr><td>Initial model used (PDB code)</td><td>Swiss-model</td><td>Swiss-model</td><td>Swiss-model</td></tr>
<tr><td>Model resolution (Å)</td><td>3.63</td><td>4.2</td><td>4.5</td></tr>
<tr><td>FSC threshold</td><td></td><td></td><td></td></tr>
<tr><td>Model resolution range (Å)</td><td>3.6-6.0</td><td>4.2-6.0</td><td>4.5-6.0</td></tr>
<tr><td>Map sharpening B factor (Å^2)</td><td>-137.6</td><td>-165.9</td><td>-244.5</td></tr>
<tr><td>Model composition</td><td></td><td></td><td></td></tr>
<tr><td>Non-hydrogen atoms</td><td>0</td><td>0</td><td>0</td></tr>
<tr><td>Protein residues</td><td>6548</td><td>2176</td><td>4848</td></tr>
<tr><td>Ligands</td><td>0</td><td>4</td><td>12</td></tr>
<tr><td>B factors (Å^2)</td><td></td><td></td><td></td></tr>
<tr><td>Protein</td><td>197</td><td>165</td><td>139</td></tr>
<tr><td>Ligand</td><td></td><td>84.9</td><td>110</td></tr>
<tr><td>R.m.s. deviations</td><td></td><td></td><td></td></tr>
<tr><td>Bond lengths (Å)</td><td>0.0113</td><td>0.0119</td><td>0.0110</td></tr>
<tr><td>Bond angles (°)</td><td>1.01</td><td>1.01</td><td>1.01</td></tr>
<tr><td>Validation</td><td></td><td></td><td></td></tr>
<tr><td>MolProbity score</td><td>1.18</td><td>1.25</td><td>1.16</td></tr>
<tr><td>Clashscore</td><td>1.00</td><td>1.95</td><td>1.42</td></tr>
<tr><td>Poor rotamers (%)</td><td>0.00</td><td>0.05</td><td>0.00</td></tr>
<tr><td>Ramachandran plot</td><td></td><td></td><td></td></tr>
<tr><td>Favored (%)</td><td>95.56</td><td>95.82</td><td>95.85</td></tr>
<tr><td>Allowed (%)</td><td>3.74</td><td>3.03</td><td>3.63</td></tr>
<tr><td>Disallowed (%)</td><td>0.70</td><td>1.15</td><td>0.52</td></tr>
</table>

Supplementary Table 2. Average main chain RMSD values (in Å) between subdomains (NBD, HD1, WHD, HD2, and LRR) of NLRP3 from Close cage (PDB ID: 7PZC), NEK7 binding (this study), open cage (this study), and activated NLRP3 inflammasome (PDB ID: 8EJ4). The RMSD values were calculated using Superpose in CCP4.

RMSD (Å)	NBD	HD1	WHD	HD2	LRR
**Close cage vs NEK7/NLRP3 dimer**	**2.31409**	**1.31659**	**1.59072**	**2.02322**	**2.07873**
**Close vs Open cages**	**2.42057**	**2.22592**	**2.26938**	**2.43403**	**2.0617**
**Activated vs Open cage**	**2.36169**	**1.52022**	**4.85262**	**2.76924**	**0.973654**
**Activated vs Close cage**	**2.94385**	**2.65039**	**4.5396**	**2.59787**	**1.67248**

Supplementary Table 3. The interface area and contact residues on the concave side of the LRR for the NEK7/NLRP3 dimer (this study), mouse closed cage (PDB ID: 7LFH), human closed cage (PDB ID 7PZC), and open octamer (this study) were calculated using PDBePISA server.

**Interface**	**NLRP3-NEK7 (This study)**	**Mus Closed cage (PDB ID: 7LFH)**	**Human Closed cage (PDB ID: 7PZC)**			**Human Open cage (This study)**	
**Contact**	NEK7-LRR	Face-Face	Face-Face	Acidic loop-Face	Total	Face-Face	Head-Face
**Interface area (Å^2)**	~2249.2	~1049.2	~724.0	~1129.6	~1853.6	~1207.1	~757.2
**Contact Residues**	74	35	22	42	64	36	26
**Overlap of Contact Residues with NEK7 Binding**	74	19	9	24	33	15	23
