# Supplementary material for: Structural basis for the oligomerization-facilitated NLRP3 activation
Source: Nat Commun. 2024 Feb 7;15:1164. doi: 10.1038/s41467-024-45396-8 (PMC10850481; doi:10.1038/s41467-024-45396-8)
Supplement: Supplementary file 3 — Description of Additional Supplementary Files [file 41467_2024_45396_MOESM3_ESM.pdf]

Description of Additional Supplementary Files

File Name: Supplementary Movie 1
Description: Cryo-EM structure of human NLRP3ΔPYD/+ATP/-MCC950 open octamer. The NACHT and LRR were colored in slate and gray, respectively.

File Name: Supplementary Movie 2
Description: Oligomerization transition from NLRP3 closed cage (PDB ID: 7LFH) to open octamer.

File Name: Supplementary Movie 3
Description: MD simulation of chimera PYD-NLRP3ΔPYD/+ATP/-MCC950 open octamer. A. Global view. B. Zoomed-in view at the Face-Face, and Back-Back interfaces. C. Zoomed-in view at the Head-Face interface reveal the putative PYD docking site at the NACHT. D. Closed view at the Tail-Tail interface. The PYD, NACHT, and LRR were in red, slate, and gray, respectively.

File Name: Supplementary Movie 4
Description: MD simulation reveals opening conformational shifts. The NBD, HD1, WHD, HD2, and LRR color-coded as in Fig. S4. NLRP3 and ATP/MCC950 were shown as cartoon, sticks and balls, respectively.
